# Mitochondrial phosphoenolpyruvate carboxykinase (PEPCK-M) and serine biosynthetic pathway genes are co-ordinately increased during anabolic agent-induced skeletal muscle growth

**DOI:** 10.1038/srep28693

**Published:** 2016-06-28

**Authors:** D. M. Brown, H. Williams, K. J. P. Ryan, T. L. Wilson, Z. C. T. R. Daniel, M. H. D. Mareko, R. D. Emes, D. W. Harris, S. Jones, J. A. D. Wattis, I. L. Dryden, T. C. Hodgman, J. M. Brameld, T. Parr

**Affiliations:** 1 School of Biosciences, University of Nottingham, Sutton Bonington Campus, Loughborough, LE12 5RD, UK; 2 School of Mathematical Sciences, University of Nottingham, University Park, Nottingham NG7 2RD, UK; 3 School of Veterinary Medicine and Science, University of Nottingham, Sutton Bonington Campus, Loughborough, LE12 5RD, UK; 4 VMRD Global Therapeutics Research, Zoetis, Kalamazoo, MI, 49007, USA

## Abstract

We aimed to identify novel molecular mechanisms for muscle growth during administration of anabolic agents. Growing pigs (Duroc/(Landrace/Large-White)) were administered Ractopamine (a beta-adrenergic agonist; BA; 20 ppm in feed) or Reporcin (recombinant growth hormone; GH; 10 mg/48 hours injected) and compared to a control cohort (feed only; no injections) over a 27-day time course (1, 3, 7, 13 or 27-days). *Longissimus Dorsi* muscle gene expression was analyzed using Agilent porcine transcriptome microarrays and clusters of genes displaying similar expression profiles were identified using a modified maSigPro clustering algorithm. Anabolic agents increased carcass (*p* = 0.002) and muscle weights (*Vastus Lateralis*: *p* < 0.001; *Semitendinosus: p* = 0.075). Skeletal muscle mRNA expression of serine/one-carbon/glycine biosynthesis pathway genes (*Phgdh*, *Psat1* and *Psph*) and the gluconeogenic enzyme, phosphoenolpyruvate carboxykinase-M (*Pck2*/PEPCK-M), increased during treatment with BA, and to a lesser extent GH (*p* < 0.001, treatment x time interaction). Treatment with BA, but not GH, caused a 2-fold increase in phosphoglycerate dehydrogenase (PHGDH) protein expression at days 3 (*p* < 0.05) and 7 (*p* < 0.01), and a 2-fold increase in PEPCK-M protein expression at day 7 (*p* < 0.01). BA treated pigs exhibit a profound increase in expression of PHGDH and PEPCK-M in skeletal muscle, implicating a role for biosynthetic metabolic pathways in muscle growth.

Homeostatic control of skeletal muscle mass is largely controlled by holding rates of protein synthesis and protein degradation in equilibrium^1^. Anabolic signals perturb this balance in favor of net protein accretion. Administration of exogenous anabolic agents, such as synthetic beta-adrenergic agonists (BA) and recombinant growth hormone (GH), are well documented to enhance skeletal muscle net protein accretion^2^,^3^. Such agents have long been of interest due to their therapeutic potential to maintain or increase muscle mass during pathological conditions whereby a loss of muscle mass negatively impacts disease progression or quality of life. Use of these agents has also been widely applied to agricultural industries striving to enhance accretion of lean mass in livestock species^2^. Whilst a plethora of academic laboratories have demonstrated the efficacious actions of BA and GH to promote lean muscle growth^4^,^5^,^6^,^7^,^8^,^9^,^10^,^11^,^12^,^13^, mechanisms underpinning this response remain unclear, thus limiting future drug discovery potential.

Research using animal models has robustly demonstrated that GH administration increases net protein accretion in skeletal muscle by increasing protein synthesis^5^,^6^,^14^,^15^. GH administration stimulates endogenous production of insulin-like growth factor 1 (IGF-1; predominantly in the liver), which is considered at least partly accountable for the anabolic effects of GH^16^,^17^,^18^. Treatment with recombinant GH activates the IGF-1-Akt-mTOR pathway^16^, which increases the translational capacity of the cell and drives protein synthesis^1^. Although signal transduction events following GH administration are well characterized in muscle, documentation of the transcriptionally regulated events remains sparse^19^.

Whilst many reports demonstrate that BA administration reduces protein degradation^8^,^9^,^13^,^20^ others have shown increased protein synthesis^20^,^21^,^22^. Accordingly, molecular analyses have identified both BA-mediated activation of protein synthetic pathways, such as the Akt-mTOR pathway^23^, and reduction of protein degradation pathways, namely repression of calpain activity and increased expression of calpastatin^20^,^24^. Mounting evidence indicates these molecular events occur in a temporal manner during BA administration^13^,^20^,^25^,^26^,^27^ highlighting the importance of time course study designs when examining these agents. Two previous studies have profiled global transcriptional events in skeletal muscle of BA treated mice and revealed extensive deregulation of gene expression^26^,^27^. However, the use of only 2 time points limits interpretation of the findings. We performed an extensive time-course (5 time points, spanning ~4 weeks) of transcriptomic profiling in skeletal muscle of pigs during administration of BA and GH to identify molecular events associated with a muscle anabolic response. Given their similarities to humans, pigs are becoming an increasingly popular animal model for biomedical research^28^, thus the information generated herein is of value to both agricultural and biomedical sciences.

## Results

### Growth performance during 27 days administration of anabolic agents

**Figure 1:**
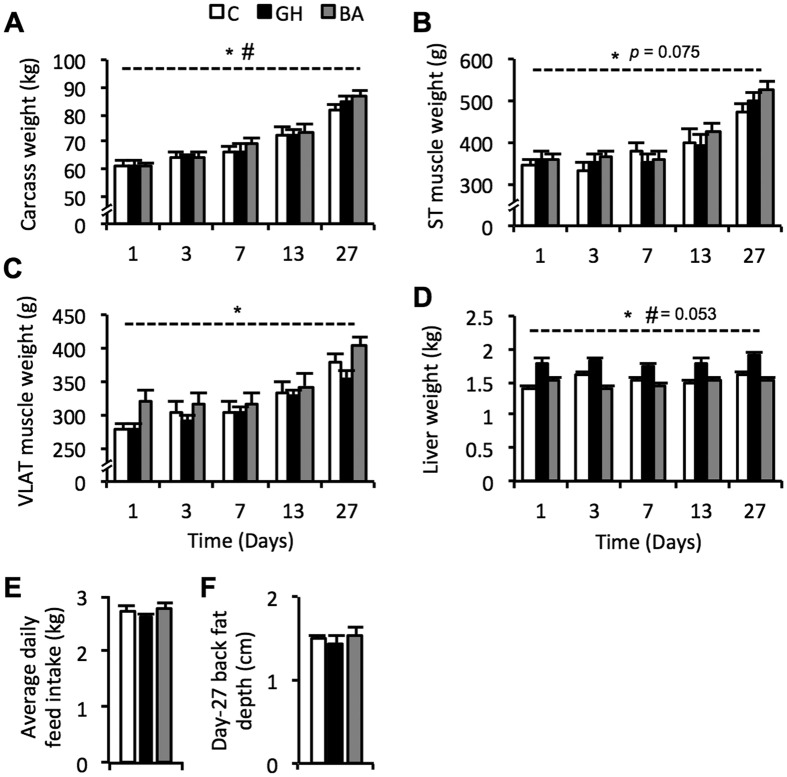
Porcine growth characteristics during 1–27 days of treatment with a beta-adrenergic agonist (grey bars; BA) or recombinant growth hormone (black bars; GH), compared to a control cohort (white bars; C). Changes in carcass weight (**A**), *Semitendinosus* (ST) muscle weight (**B**), *Vastus Lateralis* (VLAT) muscle weight (**C**) and liver weight (**D**) were measured. Average daily feed intake (**E**) and back fat depth (**F**) were measured following 27 days of treatment with beta-adrenergic agonist (BA) or growth hormone (GH) compared to a control cohort. Data is mean ± SEM. *Indicates a significant treatment effect with *p* < 0.005 (unless otherwise stated). ^#^Indicates a significant treatment x time interaction with *p* < 0.01 (unless otherwise stated).

Carcass weights displayed a significant treatment (*p* = 0.002) and treatment x time interaction (*p* < 0.001) during treatment with anabolic agents (Fig. 1A). Average carcass weight across the entire time-course was highest in pigs treated with BA (72.05 kg), whilst GH (70.77 kg) and the controls (70.5 kg) were similar (standard error of difference (SED): 0.47). Treatment with anabolic agents also caused an effect on ST and VLAT muscle weights (treatment effect: *p* = 0.075 and *p* < 0.001, respectively; Fig. 1B,C). Treatment group averages for ST and VLAT muscle weights across the 27-day treatment were highest in BA treated pigs; ST: 416.2 g for BA, 399.5 for GH, and 395.2 g for C (SED: 9.88); VLAT: 343.5 g for BA, 314.2 g for GH, and 326.3 g for C (SED: 6.84). Taken together, average carcass and muscle weights during the 27-day course of treatment were significantly higher in BA treated pigs compared to controls, whereas the growth effect of GH treatment was negligible.

We observed a consistently enlarged liver in pigs treated with GH (treatment effect: *p* < 0.001; treatment x time interaction: *p* = 0.053; Fig. 1D). The average liver weight was 1.81 kg for GH versus 1.53 kg for controls and 1.49 kg for BA (SED: 0.028). Following 27 days of treatment with anabolic agents, there was no change in back fat depth (*p* = 0.808) or daily feed intake (*p* = 0.369; Fig. 1E,F).

### Differentially expressed microarray probes with BA and GH treatment

**Figure 2:**
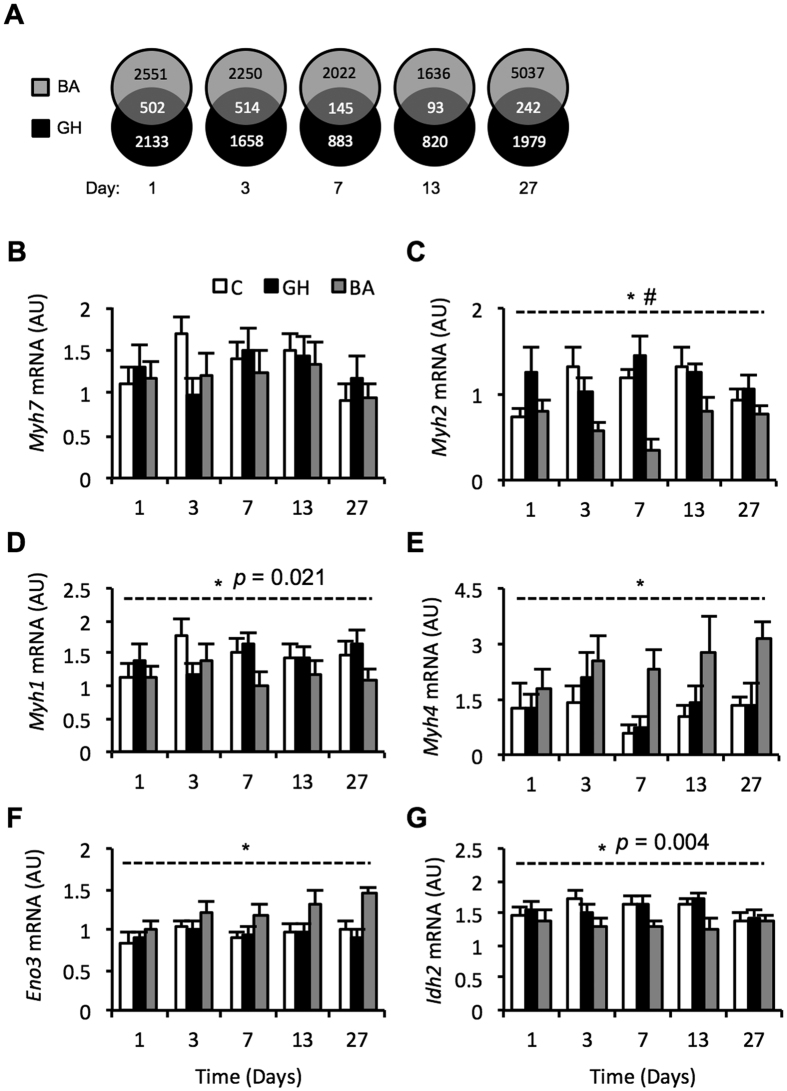
Differentially expressed microarray probes (*p* < 0.05) and changes in muscle fibre type-specific gene expression in porcine Longissimus Dorsi muscle during 1–27 days of treatment with anabolic agents. Numbers in the overlapping section of the venn diagrams represent shared differentially expressed probes by treatment with a beta-adrenergic agonist (grey circles; BA) and recombinant growth hormone (black circles; GH) (**A**). Porcine *Longissimus Dors*i muscle transcript abundance of myosin heavy chain isoform genes during 1–27 days exposure to beta-adrenergic agonist (grey bars; BA) or recombinant growth hormone (black bars; GH) treatment compared to a control cohort (white bars; C); Myosin heavy chain I: *Myh7* (**B**), Myosin heavy chain IIA: *Myh2* (**C**), Myosin heavy chain IIX: *Myh1* (**D**), myosin heavy chain IIB: *Myh4* (**E**). Metabolic genes Enolase 3: *Eno3* (**F**), and Isocitrate dehydrogenase 2: *Idh2* (**G**), were measured as indicators of glycolytic and oxidative gene expression, respectively. Data is mean ± SEM. *n* = 10 for days 1, 3, 7 and 13, whilst *n* = 15 for day 27. *Indicates a significant treatment effect with *p* < 0.001 (unless otherwise stated). ^#^Indicates a significant treatment x time interaction with *p* < 0.05.

Relative to the control group, treatment with BA caused more differentially expressed probes at all time points studied compared to GH treatment (Fig. 2A). This effect was more pronounced at the latter time points, with at least 50% fewer probes changing with GH compared to BA from day 7 onwards (Fig. 2A). BA and GH treated animals shared between 93 and 514 differentially expressed probes at any given time point during the 27 day time course, with the largest overlap occurring at days 1 and 3 (502 and 514 probes, respectively; Fig. 2A). The number of probes changing in response to BA and GH treatment decreased with time following the first day of treatment before sharply increasing again between 13 and 27 days of treatment (Fig. 2A).

### Dynamic alterations in myosin heavy chain mRNA isoform expression by BA treatment

A large number of microarray probes for myosin heavy chain (MyHC) isoforms displayed differential expression by BA treatment (*p* < 0.05). Quantification of MyHC isoform transcript abundance by Q-RT-PCR confirmed these observations (Fig. 2B–E). Dynamic alterations amongst the type II MyHC mRNA isoforms occurred with treatment (MyHC IIA (*Myh2*): *p* < 0.001, MyHC IIX (*Myh1*): *p* = 0.021 and MyHC IIB (*Myh4*): *p* < 0.001; Fig. 2C–E), with no change in expression of the MyHC I (*Myh7*) mRNA isoform (*p* = 0.57; Fig. 2B). Relative to the control, BA treatment increased MyHC IIB (*Myh4*) mRNA expression at all time points (Fig. 2E). Expression of MyHC IIA (*Myh2*) mRNA was reduced by BA treatment in four of the five time points studied, with treatment effects occurring in a time dependent manner (treatment-time interaction: *p* = 0.035; Fig. 2C). These data indicate a transition to faster MyHC mRNA isoforms with BA treatment. No alterations in MyHC mRNA isoform expression occurred during the 27 days of GH administration (Fig. 2B–E). Transcript abundance of *Eno3* and *Idh2* were used as markers of glycolytic and oxidative gene expression, respectively (Fig. 2F,G). *Eno3* and *Idh2* mRNA expression were elevated and reduced, respectively, by BA treatment relative to the controls at all time points studied (Fig. 2F,G). These findings implicate a shift in metabolic gene expression with BA but not GH treatment.

### MaSigPro clustering of differentially expressed probes: identification of amino acid metabolism genes

**Figure 3:**
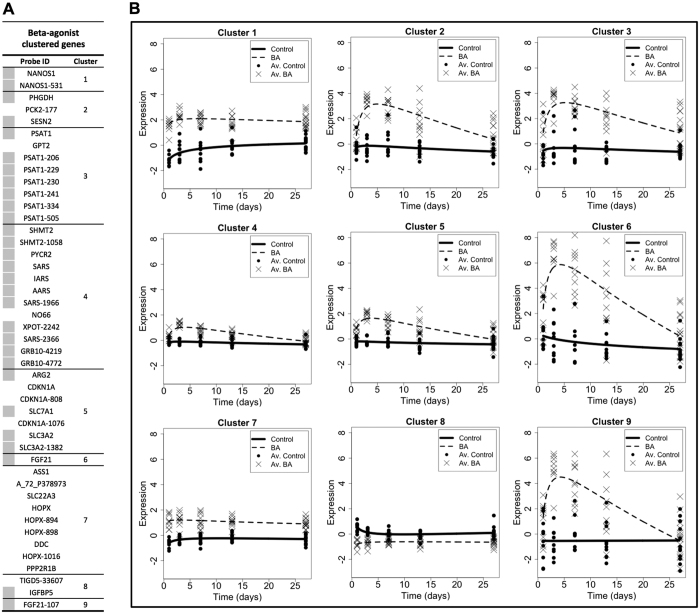
MaSigPro clustering of differentially expressed microarray probes (*p* < 0.05) generated using RNA from beta-adrenergic agonist (thin dashed line; BA) treated Longissimus Dorsi (LD) muscle, compared to non-treated controls (solid thick line). Clustering of probes was conducted using a significance of *p* < 0.05 and a stringency R^2^ value of 0.5. The table of clustered microarray probes (**A**) displays the Probe ID (with gene name if known), the cluster group, and a grey square if that probe was also clustered in the equivalent analysis for growth hormone treated samples (see Fig. 4). The 9 clustered probe plots (**B**) display time on the x-axis (spanning the treatment duration of 1–27 days) with relative expression on the y-axis. Each plot depicts the expression profile of the microarray probes within that cluster for beta-adrenergic agonist (BA) treated and non-treated control animals. *n* = 10 for days 1, 3, 7 and 13, whilst *n* = 15 for day 27. Av. indicates average.

**Figure 4:**
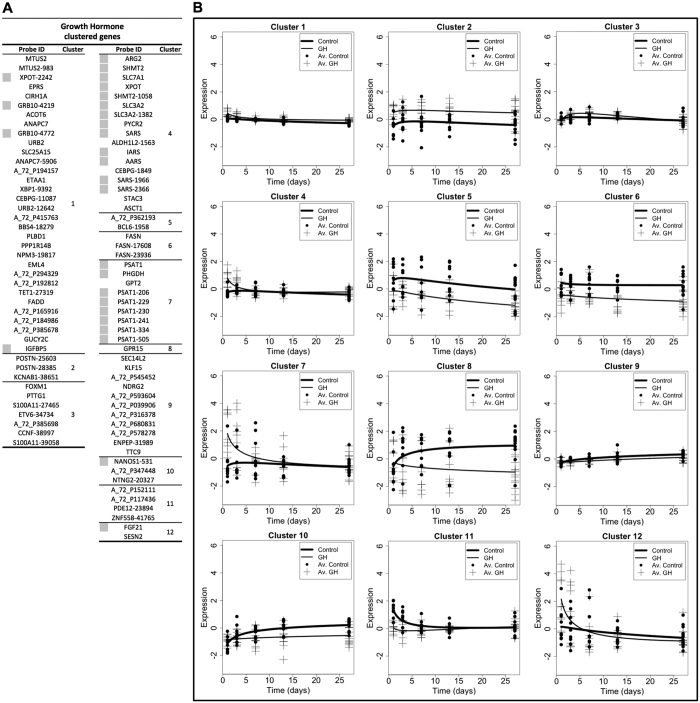
MaSigPro clustering of differentially expressed microarray probes (*p* < 0.05) generated using RNA from growth hormone (solid thin line; GH) treated Longissimus Dorsi (LD) muscle, compared to non-treated controls (solid thick line). Clustering of probes was conducted using a significance of *p* < 0.05 and a stringency R^2^ value of 0.2. The table of clustered microarray probes (**A**) displays the Probe ID (with gene name if known), the cluster group, and a grey square if that probe was also clustered in the equivalent analysis for beta-adrenergic agonist treated samples (see Fig. 3). The 12 clustered probe plots (**B**) display time on the x-axis (spanning the treatment duration of 1–27 days) with relative expression on the y-axis. Each plot depicts the expression profile of the microarray probes within that cluster for growth hormone (GH) treated and non-treated control animals. *n* = 10 for days 1, 3, 7 and 13, whilst *n* = 15 for day 27. Av. indicates average.

To identify novel gene targets associated with growth promoter administration, we did not use conventional pathway or gene ontology analyses. Instead, we utilized a mathematical clustering approach to identify groups of differentially expressed probes/genes based on their pattern and magnitude of change in response to treatment and time. Probes from each treatment were independently clustered against the controls (BA versus control; GH versus control). Using a stringency R^2^ value of 0.5, a subset of differentially expressed probes clustered into 9 groups for BA (Fig. 3) and zero groups for GH. Due to a low magnitude of change and concurrent variability amongst probes from the GH treated group, a lower stringency R^2^ value of 0.2 was required to generate a similar number of clusters to that observed with BA (Fig. 4). This approach yielded 12 clusters of differentially expressed probes for the GH treated group, albeit with less well-fitted regression curves (Fig. 4B). Accordingly, these clusters revealed a generally lower magnitude of change in gene expression by GH treatment in comparison to that induced by BA treatment. Furthermore, it is noteworthy that almost 78% of the clustered probes for the BA treatment group appeared in the top 50 most significantly changed probes at day 3, highlighting that BA clustered probes were amongst the most profoundly changing genes at this time point. In contrast, only 11 and 7% of the GH clustered probes appeared in the top 50 most significantly changed probes for GH at days 1 and 3 respectively. Therefore, clustering of differentially expressed probes from GH treated pigs revealed only weak time-dependant alterations in gene expression with a low magnitude of change, whereas BA treated pigs revealed extensive temporal changes in gene expression that represented a cohort of genes with a large magnitude of change. This may represent differential potency of BA and GH to induce direct alterations in skeletal muscle gene expression.

Despite differences in the magnitude of change by clustered probes in BA and GH treated groups, a high number of differentially expressed probes, targeting 16 different genes, were clustered in both treatment groups (all increasing relative to the control cohort; highlighted in grey in Figs 3A and 4A). Many of these overlapping genes encoded proteins involved in amino acid metabolism such as: serine/glycine/proline biosynthesis (*Phgdh*, *Psat1*, *Shmt2*, *Pycr2*), tRNA synthetases (*Sars*, *Iars*, *Aars*), or tRNA and amino acid transporters (*Xpot*, *Slc7a1*, *Slc3a2*). Probes detecting transcripts for the growth factor, *Fgf21*, and growth factor binding protein, *Grb10*, were also clustered by BA and GH treatment, both displaying an increase with treatment. Compared to all other differentially expressed probes in BA clustered groups, *Fgf21* probes displayed the most pronounced increase and were consequently singularly “clustered” independently of any other probes (Fig. 3B; BA clusters 6 and 9). A number of probe clusters generated for BA and GH treatments also contained multiple probes for the same gene. The most notable recurring probes were those for *Psat1* transcripts, with 7 *Psat1* probes within a distinct cluster displaying increased expression of this gene in both BA and GH groups (BA cluster 4 (Fig. 3B); GH cluster 7 (Fig. 4B)). This indicated that increased gene expression of *Psat1* was potentially the strongest common response across BA and GH treated groups (which led to later validation by Q.RT.PCR).

Not only was there an overlap in the individual probes identified by cluster analysis for both BA and GH treatment groups, many of these probes were similarly grouped by both treatments (BA clusters 3 and 4 (Fig. 3B); GH clusters 4 and 7 (Fig. 4B)). However, the temporal expression profiles of these similarly grouped probes were distinct during treatment with BA and GH. For instance, BA induced a tightly controlled and prolonged elevation in expression of probes in BA clusters 3 and 4 (Fig. 3B), with expression peaking around day 3 and gradually returning towards control levels by day 27, whereas GH induced only a modest, highly variable and transient increase of these same probes (in GH clusters 4 and 7 (Fig. 4B)), peaking on day 1 of treatment and returning to control levels from day 7 onwards. Therefore, both BA and GH treatment co-ordinately regulated similar groups of genes involved in amino acid metabolism but the resulting temporal expression and magnitude of change were dependent on the treatment administered.

### Validation of gene targets reveals potency of BA compared to GH on metabolic gene regulation

**Figure 5:**
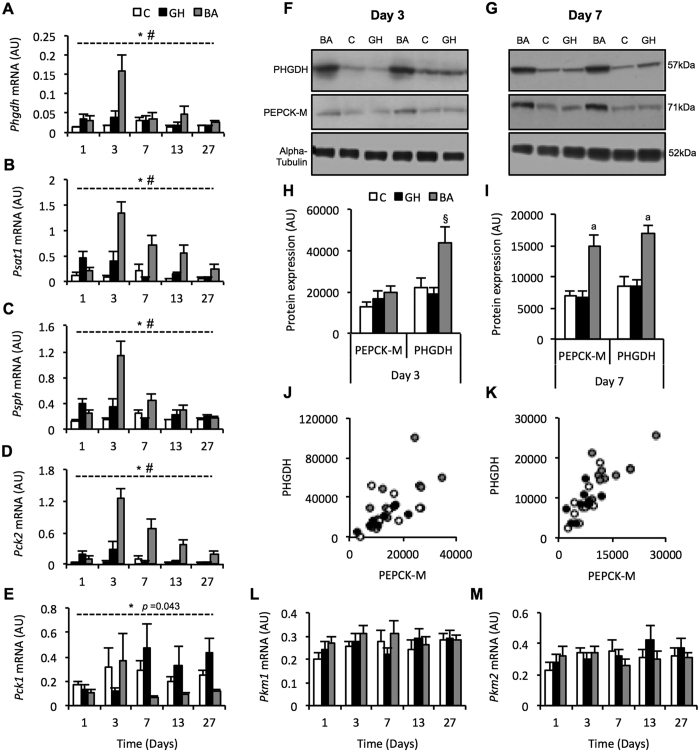
Validating expression of enzymes involved in the biosynthesis of anabolic intermediates to confirm genes that were identified as differentially expressed in the maSigPro clustering analysis. Porcine *Longissimus Dors*i muscle transcript abundance of the following genes was determined by quantitative-RT-PCR to establish the effects of a beta-adrenergic agonist (grey bars; BA) or recombinant growth hormone (black bars; GH) treatment for 1–27 days, compared to a control cohort (white bars; C); Phosphoglycerate Dehydrogenase: *Phgdh* (**A**), Phosphoserine aminotransferase: *Psat1* (**B**), Phosphoserine Phosphatase: *Psph* (**C**), the mitochondrial isoform of phosphoenolpyruvate carboxykinase: *Pck2* (**D**), the cytosolic isoform of phosphoenolpyruvate carboxykinase: *Pck1* (**E**), the muscle isoform of Pyruvate Kinase variant 1: *Pkm1* (**L**), and Pyruvate Kinase variant 2, *Pkm2* (**M**). Representative western blots (F,G) and quantification (H,I) of beta-adrenergic agonist (BA) and growth hormone (GH) treatment effects on *Longissimus Dors*i (LD) muscle protein expression of Phosphoglycerate Dehydrogenase: PHGDH, and mitochondrial isoform of phosphoenolpyruvate carboxykinase: PEPCK-M, following 3 (**F,H**) and 7 (**G,I**) days of treatment. Alpha-tubulin was used as a loading control. Relationship between PHGDH and PEPCK-M protein expression in porcine *Longissimus Dors*i muscle following treatment with a beta-adrenergic agonist (grey circles), growth hormone (black circles) or no treatment (white circles) following 3 (**J**) and 7 (**K**) days. Data is mean ± SEM. *n* = 10 for days 1, 3, 7 and 13, whilst *n* = 15 for day 27. *Indicates a treatment effect with *p* < 0.001 (unless otherwise stated). ^#^Indicates a treatment x time interaction with *p* < 0.001. a Indicates a treatment effect with *p* < 0.01. ^§^Indicates a treatment effect with *p* < 0.05.

Clustering of microarray data using maSigPro revealed that both BA and GH treatment coordinately increased expression of genes involved in amino acid metabolism. Accordingly, transcript abundance of a subset of these genes was examined by quantitative PCR to affirm the temporal expression profiles induced by BA and GH treatments (Fig. 5A–D).

The cluster analysis identified that genes encoding the initial two enzymes in the serine/one-carbon/glycine (SOG) biosynthesis pathway (*Phgdh* and *Psat1*) were increased by BA and GH treatment. We examined mRNA expression of SOG biosynthesis pathway enzymes (*Phgdh*, *Psat1* and *Psph*) and revealed significant treatment and treatment x time effects for all three genes (*p* < 0.001; Fig. 5A–C). BA treatment induced a robust and similar temporal increase in mRNA expression of *Phgdh*, *Psat1* and *Psph*, with the largest increase relative to controls occurring on day 3. The initiating enzyme of the SOG biosynthesis pathway, PHGDH, also displayed an approximately 2-fold increase in protein expression with BA treatment at days 3 and 7 (*p* = 0.010 and *p* < 0.001, respectively for ANOVA; Fig. 5F–I). Interestingly, baseline transcript abundance for *Phgdh*, *Psat1* and *Psph* in porcine skeletal muscle (*n* = 10) was low (crossing point values: 40, 32.4 and 30.2, respectively), indicating that BA-mediated induction of SOG biosynthesis pathway genes (particularly *Phgdh*) could have profound biosynthetic consequences.

GH treatment had no effect on PHGDH protein expression at days 3 or 7 (*p* > 0.05 for both Dunnett’s tests; Fig. 5F–I), but consistent with the maSigPro clustering analysis, GH treatment did elevate *Phgdh*, *Psat1* and *Psph* mRNA transcripts above controls at days 1 and 3 (Fig. 5A–C), just not to the same magnitude as BA.

**Figure 6:**
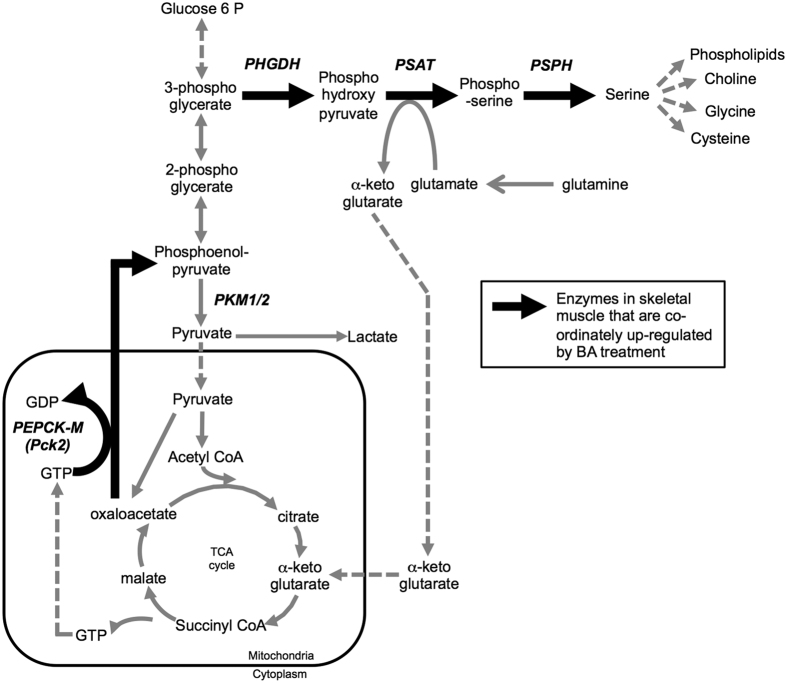
A schematic illustrating the relationship between glycolysis, the TCA cycle and pathways associated with serine/one-carbon/glycine (SOG) biosynthesis. Bold black arrows indicate enzymes that were up-regulated at the mRNA (*Pck2*, *Phgdh*, *Psat1*, *Psph*) and/or protein level (PEPCK-M and PHGDH) by beta-adrenergic agonist treatment.

Given the robust, coordinated up-regulation of multiple transcripts encoding SOG biosynthesis enzymes by BA treatment (see schematic in Fig. 6), we next assessed expression of the mitochondrial enzyme, PEPCK-M (encoded by the *Pck2* gene), which was also identified by the maSigPro clustering analysis (Fig. 3A; BA cluster 2). PEPCK-M generates phosphoenolpyruvate (PEP) from oxaloacetate, which can feed into gluconeogenesis and subsequently biosynthetic pathways, such as the SOG biosynthesis pathway^29^,^30^,^31^ as illustrated in the schematic in Fig. 6. *Pck2* mRNA expression displayed a strong treatment and treatment x time interaction (*p* < 0.001; Fig. 5D). Similar to the SOG pathway genes, *Pck2* transcript abundance displayed marked elevation by BA, with the largest response occurring at day 3 and remaining elevated above control levels through to day 27. Expression of the *Pck2* encoded protein, PEPCK-M, was increased approximately 2-fold by BA treatment at day 7 (p < 0.001 for ANOVA; Fig. 5G,I), but there was no effect at day 3 (*p* = 0.245 for ANOVA; Fig. 5F,H). GH treatment induced a modest and transient increase in *Pck2* mRNA expression at days 1 and 3 (Fig. 5D), with no corresponding changes in PEPCK-M protein expression at days 3 or 7 (*p* = 0.245; Fig. 5F–I).

A scatter plot depicting PEPCK-M and PHGDH protein abundance at day 7 clearly shows that individual pigs treated with BA displayed elevated expression of both PEPCK-M and PHGDH (Fig. 5K; *r* = 0.81). This effect was less apparent at day 3 but still evident in some BA treated pigs (Fig. 5J; *r* = 0.64). Transcript abundance of the cytosolic form of PEPCK, PEPCK-C (encoded by the *Pck1* gene), showed high variability and no consistent effects of BA and GH treatment (treatment: *p* = 0.043; treatment x time interaction: *p* = 0.103; Fig. 5E). It is worth noting that the transcript abundance of *Pck1* and *Pck2* in porcine skeletal muscle was very low in the controls (crossing point values: 36.06 and 35.83, respectively), and that the BA-mediated induction of the *Pck2* gene and its encoded protein, PEPCK-M, represents a substantial perturbation of the homeostatic control of *Pck2* expression in adult skeletal muscle.

Pyruvate kinase splice variants 1 and 2 (PKM1/2) irreversibly convert PEP to pyruvate with differing degrees of efficiency^32^,^33^. Increased expression of the PKM2 splice variant (at the expense of the PKM1 variant) has been shown to increase the diversion of glucose carbons into biosynthetic pathways, such as the SOG biosynthesis pathway^32^,^33^,^34^,^35^. We measured *Pkm1* and *Pkm2* splice variant mRNA expression to reveal no change in response to BA and GH treatment (*p* = 0.365 (*Pkm1*) and *p* = 0.515 (*Pkm2*); Fig. 5L,M).

Finally, *Fgf21* probes were clustered by both BA and GH treatment groups but showed a particularly pronounced increase by BA treatment (Fig. 3B; BA clusters 6 and 9). Due to the very low level of *Fgf21* mRNA expression in porcine skeletal muscle, it was not possible to generate a standard curve for relative quantification. Instead, crossing point (Cp) values are presented, with a decrease in Cp value indicating an increase in mRNA expression, which illustrates that BA, but not GH, induced *de novo* expression of the *Fgf21* transcript (Supplementary Fig. S2). This was confirmed by end-point PCR amplification and subsequent visualization of a larger fragment of the *Fgf21* transcript at day 3 (see gel image in Supplementary Fig. S2). We attempted to measure FGF21 protein expression by western blot but were unable to produce reliable measurements due to the lack of a commercially available reliable antibody against porcine FGF21.

## Discussion

Using extensive time-course transcriptomic profiling of porcine skeletal muscle, we aimed to identify molecular events induced by the anabolic agents, Ractopamine (a beta-adrenergic agonist; BA) and Reporcin (recombinant porcine growth hormone; GH). Since the control group was not injected, it is possible, albeit unlikely, that the injection *per se* contributed to the GH-mediated effects observed herein. However, the biggest effects reported herein were observed in the BA treated group, for which the feed only control cohort was entirely appropriate. Our analysis revealed extensive changes in expression of genes involved in amino acid metabolism during administration of these anabolic agents. Most notably, we identified that BA treated pigs exhibit a previously unreported increase in protein expression of the PHGDH and PEPCK-M enzymes in skeletal muscle. The physiological role of these enzymes in skeletal muscle warrants further exploration.

### Growth performance

Despite the wide spread commercial use of BA and GH by pig production industries in the USA and Australia, we report only modest (but significant) improvements to carcass and muscle growth with these agents. We and others^36^ suspect that extensive genetic selection for fast growing pig breeds^37^, such as the one used herein, has limited their sensitivity to exogenous anabolic agents. Improvements in growth rate by anabolic agents in pigs is variable^12^,^36^ and the improvements reported herein are in line with the expected response^12^,^36^. We also checked for other biological/physiological hallmarks of a BA or GH response. For instance, we observed the well-documented transition in MyHC isoform gene expression with BA treatment, shifting towards a persistently elevated expression of the fast type IIB mRNA isoform^38^,^39^,^40^. Treatment with GH induced a sustained elevation in liver weight (through days 1–27), a typical response to exogenous GH administration^18^,^38^. Together, these responses confirmed efficacious delivery of the anabolic agents.

### Extensive temporal differential expression of genes involved in amino acid metabolism and transport

The major finding from the cluster analysis of BA- and GH-induced differential gene expression was an extensive up-regulation of transcripts encoding enzymes involved in several aspects of amino acid metabolism. These pathways included serine/glycine/proline biosynthesis (*Phgdh*, *Psat1*, *Shmt2*, *Pycr2*), tRNA synthetases (*Sars*, *Iars*, *Aars*), as well as tRNA and amino acid transporters (*Xpot*, *Slc7a1*, *Slc3a2*). This response was more pronounced for treatment with BA than GH, with expression profiles showing a more sustained response with a greater magnitude of change. Previous transcriptomic studies using rodents treated with BA have also identified significant alterations in metabolic gene expression^26^,^27^ but have not reported the coordinated alterations in the amino acid metabolic pathways identified herein. This could be due to species-specific differential response to BA, differences in the class of BA used, the sensitivity of the transcriptomic technologies employed, or the time points selected for transcriptomic profiling. We observed the largest increase in expression of amino acid metabolism related genes by BA at day 3, a time-point not used by previous studies (ref. 26: days 1 and 10; ref. 27: hours 1 and 4) and therefore the most likely reason for this response not being previously reported. The time-dependent alteration in skeletal muscle gene expression identified by our cluster analysis demonstrates the temporal transcriptional plasticity of skeletal muscle to an exogenous stimulus.

We decided to focus on genes involved in serine/one-carbon/glycine (SOG) biosynthesis (*Phgdh*, *Psat1* and *Psph*) because of the recent explosion of interest in this pathway to facilitate growth in rapidly proliferating cells^41^,^42^,^43^,^44^,^45^, with no previous reports of a role in adult skeletal muscle.

### Co-ordinated regulation of SOG biosynthesis pathway genes by BA and GH administration

We identified a previously unreported increase in gene expression of three consecutive enzymes in the SOG biosynthesis pathway (*Phgdh*, *Psat1* and *Psph*) in skeletal muscle of BA treated pigs. Elevated expression of these genes is typically restricted to highly proliferative cancer cells^41^,^42^,^43^ and proliferating embryonic stem cells^44^, with expression of these genes coordinately diminishing as cells differentiate^44^. Unpublished observations from our laboratory also show reduced expression of SOG pathway genes during muscle cell differentiation *in vitro*. Therefore, the BA- (and to a lesser extent, GH-) mediated increases in gene expression of SOG pathway genes in a terminally differentiated adult skeletal muscle (presented herein) were somewhat surprising results. BA treatment induced a 2-fold increase in protein expression of PHGDH, lasting at least 5 days. PHGDH catalyzes the first reaction in the SOG biosynthesis pathway, converting 3-phosphoglycerate (3-PG) to phosphohydroxypyruvate, effectively shunting glucose carbons away from glycolysis and into macromolecule biosynthesis^32^. A role for PHGDH has not previously been defined in skeletal muscle, but the SOG biosynthesis pathway produces a number of anabolic intermediates, such as serine, glycine, cysteine and phospholipids^41^,^43^,^44^,^46^, which are essential for protein synthesis and/or cell growth. Functional genomics has revealed that PHGDH is a fundamental regulator of SOG pathway flux and cell proliferation^41^,^43^,^45^,^46^. Overexpression of PHGDH in non-tumorigenic cells increases SOG pathway flux^46^, whilst knockdown of PHGDH in breast and lung cancer cells reduces flux through this pathway^41^,^43^.

Hyperactivity of the SOG pathway, induced by elevated expression of PHGDH or PSPH, confers a significant growth advantage in many proliferating cell types^41^,^43^,^45^,^46^. The identification of a coordinated increase of SOG pathway genes by BA treatment, and to a lesser extent GH treatment (presented herein), is novel and suggests this pathway may also yield anabolic intermediates to support the growth of terminally differentiated muscle, which warrants further exploration. Sustained treatment with a BA is well documented to impair oxidative metabolism in skeletal muscle and induce a more fast-contracting, glycolytic phenotype^38^,^40^,^47^,^48^. Yet it has remained unknown as to how or why this metabolic shift, from oxidative to glycolytic, may be instrumental in BA-induced muscle hypertrophy. A switch to a glycolytic metabolism in cancer cells occurs not to rapidly supply ATP but to support increased biosynthesis of molecules required for cell growth^32^. The BA-mediated switch to a more glycolytic phenotype may also occur to support a requirement for increased diverted flux of glucose carbons through biosynthetic pathways, as indicated by the coordinated increase in expression of SOG pathway genes presented herein. Functional studies are needed to test this hypothesis.

Serine is a nutritionally non-essential amino acid, but is considered metabolically indispensable^31^, serving as an intermediate for many cellular processes. Treatment with a BA may increase reliance on SOG pathway derived products such as serine and glycine, potentially for protein synthesis^20^,^23^, thus increasing expression of SOG pathway enzymes to meet this demand. The coordinated increase in SOG pathway gene expression in BA and GH treated animals may also indicate that serine (and glycine) become limiting following administration of anabolic growth promoters. 

The amino acids, serine and glycine, are not the only end products of the SOG biosynthesis pathway. Metabolomic analysis following knockdown of PHGDH in rapidly growing cells reveals a dramatic reduction in alpha-ketogluterate (aKG). The transaminase, PSAT1, regulates the second step in the SOG pathway and produces aKG from glutamate. aKG can restore TCA cycle flux^31^,^41^,^44^,^49^ or be used as a precursor for synthesizing other amino acids such as proline, arginine and glutamate^32^,^49^. Based on the data generated herein, functional studies are required to examine a) whether BA’s divert carbon flux into the SOG biosynthesis pathway and b) examine the physiological implications of increased PHGDH levels *per se* on skeletal muscle mass and function.

### Increased expression of PEPCK-M (Pck2) by BA implicates gluconeogenic flux in skeletal muscle growth

We report that BA treatment increased skeletal muscle expression of PHGDH, an enzyme capable of controlling carbon flux into the SOG biosynthesis pathway in rapidly growing cells^41^,^42^,^43^. We searched our list of BA clustered genes for metabolic enzymes that could divert carbon flux towards the SOG pathway. PEPCK-M (encoded by *Pck2*) converts mitochondrial oxaloacetate to phosphoenolpyruvate (PEP) and thereby supplies carbons from TCA cycle intermediates into gluconeogenic and biosynthetic pathways^29^,^30^,^31^. The robust increase in PEPCK-M mRNA and protein expression by BA treatment was considered biologically interesting given the very low or complete lack of expression of PEPCK-M transcripts or protein in adult muscle (ref. 50 and this study).

There are two PEPCK isozymes, a cytoplasmic form, PEPCK-C (encoded by *Pck1*), and the mitochondrial form, PEPCK-M (encoded by *Pck2*). Although the role of PEPCK-C has been thoroughly characterized in liver for its role in gluconeogenesis, its counterpart isozyme, PEPCK-M, has remained astonishingly under explored^30^. Recent reports, however, revealed a role for PEPCK-M in supporting tumour growth and cell proliferation, particularly in metabolically unfavorable environments^29^,^51^,^52^. Carbon labeling studies have demonstrated that knockdown of PEPCK-M or use of the PEPCK inhibitor, MPA, inhibits incorporation of oxaloacetate-derived carbons into serine and glycine^29^. This implicates PEPCK-M as an enzyme capable of redirecting TCA cycle intermediates into the SOG biosynthesis pathway^29^. The findings presented herein that BA treatment coordinately up-regulates expression of PEPCK-M and the first enzyme in the SOG biosynthesis pathway, PHGDH, is therefore particularly interesting. This coordinated response provides strong rationale for future studies to explore the role of PEPCK-M and PHGDH in diverting metabolic flux towards increasing biosynthetic outputs in terminally differentiated skeletal muscle.

### Expression of Fgf21 mRNA in porcine skeletal muscle by BA treatment

The most profoundly changing probe in the maSigPro clustering analysis for BA and GH treatment was for the Fibroblast Growth Factor 21 gene, *Fgf21*. Analysis of *Fgf21* transcript abundance in porcine skeletal muscle revealed very low basal expression and that BA treatment induced expression of *Fgf21* mRNA expression following 3 days of treatment. We were unable to monitor whether this change at the transcript level resulted in an increase in FGF21 protein expression due to a lack of reliable antibodies detecting porcine FGF21. Recent studies implicate FGF21 as a modulator of insulin sensitivity and glucose uptake^53^, and also as a sensor of protein restriction^54^, suggesting this growth factor may facilitate compensatory metabolic functions. Further studies are required to examine whether BA treatment influences skeletal muscle FGF21 expression at the protein level, as well as the functional significance of any transient increase in skeletal muscle expression.

## Conclusion

For the first time, we show that BA treated pigs exhibit increased expression of PHGDH and PEPCK-M in skeletal muscle. These enzymes are known to regulate flux of glucose carbons into biosynthetic pathways for the generation of anabolic intermediates. We are now conducting studies to establish whether PHGDH and PEPCK-M are capable of driving or modulating a muscle anabolic response.

## Materials and Methods

### Experimental design

Pigs administered with a beta-adrenergic agonist (BA) or recombinant growth hormone (GH) were compared to a control cohort over a 27-day time course (1, 3, 7, 13 and 27 days) to elucidate molecular events associated with acute and sustained exposure to muscle anabolic agents.

There is no sham-injected control for the GH treatment included in this study. This study examined the effects of 2 treatments (BA and GH), which includes both the agents used and the associated administration practices used commercially. Therefore the commercial use of Reporcin (Growth Hormone) includes the injection and this is considered part of the treatment.

### Time course of growth promoter administration

Approximately 5-month-old growing gilts (Duroc x (Landrace x Large White); C23 × 337 progeny; purchased from PIC, UK), with a mean starting weight of 77 kg (±7 kg), were obtained in 3 batches (*n* = 51, 51 and 63, respectively), individually housed and allowed to acclimatise in the Bio-Support Unit at The University of Nottingham for 7 days. Pigs were fed twice daily for 45 minutes on a non-pelleted, 16.7% protein finisher feed (14 MJ/kg; Target Feeds, UK) throughout the entirety of the study. Calculations for predicted *ad libitum* feed intake were based on the Akey established feed intakes for acceptable swine performance for standard genotypes (Brookville, OH, USA; a commercial pig feed supplier) along with estimations of feed intake derived from Chaosap and colleagues^55^. Using these figures, pigs were provided with 30% more feed than that predicted based on their body weight, to mimic *ad libitum*-like feeding. Feed quantities were adjusted based on body weight measurements taken at the start of each week and the feed quantity remained unchanged for the duration of that week. Refused feed was measured to determine actual feed intake.

Following acclimatisation, pigs were provided with feed containing Ractopamine HCL (20 ppm; Elanco Animal Health, Grenfield, IN) or administered an intramuscular injection (approximately 70 mm behind the base of the ear) of recombinant growth hormone (10 mg/pig/48 hours; Reporcin, Zamira), for 1, 3, 7, 13 (*n* = 10 per treatment group per time point) or 27 days (*n* = 15 per treatment group). Comparisons were made against a control cohort that received feed only (no injections). Treatment doses were selected based on doses currently used in commercial pig production in the USA (Ractopamine) and Australia (Reporcin). Allocation of treatment and time cohorts was conducted as follows: after initial weighing, pigs were allocated in groups of 3 to adjacent pens, based on similar initial body weights and then treatments were assigned randomly within those groups of 3, with each group of 3 randomly assigned to a time point. This was done to ensure equivalent starting weights per treatment and time point, with pigs subsequently slaughtered in the groups of 3 (one for each treatment) at the appropriate time point. Body weights of treatment and time allocated cohorts were not different at the start of the intervention (Supplementary Fig. S1). The time and/or treatment allocations were blinded from all staff undertaking animal husbandry, during the slaughter and tissue collection, and during the molecular analyses (of RNA and protein). The total number of pigs completing the trial was 164 (1 GH-treated pig was removed from the trial due to illness unrelated to the treatment). This project was approved by the University of Nottingham Ethical Review Committee and was carried out in accordance with the UK Animals (Scientific Procedures) Act of 1986 (Project Licence PPL 40/3010).

### Tissue collection and analysis at slaughter

Pigs were electrically stunned prior to exsanguination and confirmed dead by a licenced slaughter man. A sample of the *Longissimus Dorsi* (LD) muscle was then isolated (from the 10^th^ rib in each pig), immediately snap-frozen in liquid nitrogen and later stored at −80 °C for RNA and protein analysis. The LD muscle was used for RNA analysis because it is reproducibly accessible within seconds after death, providing the most highly representative RNA sample of live skeletal muscle. Following removal of the visceral organs, the carcass weight was determined. The *Semitendinosus* (ST) and *Vastus Lateralis* (VLAT) muscle was then dissected and weighed as an indicator of change in muscle growth. The ST and VLAT muscles have a discrete origin and insertion (unlike the LD), making them suitable candidate muscles for whole muscle dissection and measurement. Following halving of the carcass, back fat depth was measured (at the 5^th^ lowest rib) using a line ruler.

### Microarray transcriptomic profiling

Total RNA was extracted from a 100 mg sample of LD muscle using TRIZOL reagent (Invitrogen, Paisley, UK) according to manufacturer instructions, followed by DNase treatment (Promega, Southampton, UK). Total RNA from all 164 pigs (5 time points, 3 treatment groups) was processed using Agilent Technologies one-color Low Input Quick Amp Labelling Kit according to the manufacturer protocol. Cyanine 3-CTP labelled cRNA was quantified using the NanoDrop 8000 (Thermo Scientific) and 1.65 μg of cRNA was then hybridized onto Agilent Technologies porcine gene expression (V2) 4 × 44 k microarrays at 65 °C for 17 hours in a SciGene Model 777 Microarray Oven. Arrays were processed in a SciGene NoZone Workspace, to limit exposure to ozone, and washed and dried using the Little Dipper^TM^ Microarray Processor, Model 650C. Arrays were scanned with the Agilent Technologies DNA Microarray C Scanner at a resolution of 5 μm.

### Modified maSigPro clustering of microarray data

Given the extensive time-course microarray data set (totalling 164 samples), we applied a clustering algorithm to the differentially expressed microarray probes to sort probes into distinct clusters. Biologically relevant expression profiles of these probes were determined using an R^2^ coefficient, which places a value on how well a curve fits the data. BA and GH treated groups were independently assessed against the controls. The approach employed herein was built upon a clustering algorithm described by Conesa and colleagues^56^ called maSigPro. To avoid giving excessive weighting to the latter time points (days 13 and 27), we used a transformed time scale, with the *j*th time point (*j* = 1, 2, 3, 4, 5) given by *T*_*j*_ = log(*t*_*j*_). Furthermore, we used a k-means clustering algorithm, which generates *k* clusters but we expanded on MaSigPro to determine an optimal value of *k*. A detailed description of our modifications to the maSigPro method is in preparation for publication in a mathematics journal and will be made available at arxiv.org.

### Quantitative and end-point PCR

Expression profiles of a subset of probes identified by maSigPro clustering of microarray data were validated by measuring the relative abundance of the respective mRNA transcripts using quantitative reverse transcriptase PCR (Q-RT-PCR). First strand cDNA synthesis was conducted on 500 ng RNA using a cDNA synthesis kit (Transcriptor First Strand cDNA Synthesis Kit, Roche, Burgess Hill, UK) according to manufacturer instructions. Q-RT-PCR was performed in duplicate on 384 well plates using a Lightcycler 480 (Roche, Burgess Hill, UK). All samples were blind labelled until final analysis of the expression data. Details of the Q-RT-PCR were described previously^57^. Relative transcript abundance was calculated using the standard curve method and expression values were normalized to total cDNA in the PCR reaction using the established oligreen method^58^, as previously described^57^. End-point PCR of the *Fgf21* transcript and subsequent visualization on a 1% agarose gel was conducted to confirm induction of *Fgf21* mRNA expression. All oligonucleotide sequences are presented in Table 1. Primer sequences for myosin heavy chain isoforms were obtained from Wimmers *et al*.^59^.

### Western blotting

Total protein was extracted from frozen day 3 and 7 treated (BA and GH) and control LD muscle samples. The extraction, immunoblotting and detection procedures were performed as previously described^60^, using ECL select (GE Life Sciences). Antibodies for PEPCK-M, PHGDH and Alpha-tubulin were purchased from Cell Signaling (#6924), Sigma Aldrich (HPA021241) and Cell Signaling (#2144), respectively.

### Statistical analyses

All statistical analyses were performed using Genstat (13^th^–15^th^ edition) and significance was accepted if *p* < 0.05. Animal growth performance data were analysed for treatment effects (BA, GH or control), time (1, 3, 7, 13 or 27 days) and treatment x time interactions by two-way analysis of variance (ANOVA), with blocking for batch of pigs. Similarly, relative changes in mRNA transcript abundance were assessed for significant treatment, time and treatment x time interactions using two-way ANOVA (no blocking). Protein expression of PEPCK-M and PHGDH at days 3 and 7 were analysed separately for each protein and day by one-way ANOVA. When appropriate (i.e. no treatment x time interactions), post-hoc Dunnett’s tests were used to compare BA and GH group means to the control group. All data are presented as mean ± standard error of the mean.

Statistical analyses built into the maSigPro clustering package employed an ANOVA to filter differentially expressed probes based on *p* < 0.05.

## Additional Information

**How to cite this article**: Brown, D. M. *et al*. Mitochondrial phosphoenolpyruvate carboxykinase (PEPCK-M) and serine biosynthetic pathway genes are co-ordinately increased during anabolic agent-induced skeletal muscle growth. *Sci. Rep*. **6**, 28693; doi: 10.1038/srep28693 (2016).

## Supplementary Material

Supplementary Information

## Figures and Tables

**Table 1:** Oligonucleotide primer sequences used for quantitative-RT-PCR.

Gene	Forward primer	Reverse primer	Reference	Notes
*Pck2*	ATCCGAAAGCTCCCCAAGTAC	CAATCACCGTCTTGCTTTCTACTC		
*Pck1*	CTGAGCCACATGGAGGAAGAG	GATACCGTCTTGCTTTCAATTC		
*Phgdh*	CTGGCCGGCGTTGTAAAC	GCTTCAGCCAGACCAATCCA		
*Psat1*	CAAAGTGCAGGCTGGAAATAACT	CCCCGCCGTTGTTCTTAA		
*Psph*	GGGCATAAGGGAGCTGGTAAG	GACGGGATGTTGAGCTTTGAA		
*Fgf21*	TCCAGACATCCCGGTTCCT	AAACGTTGTAGCCATCCTCAAGA		Q.RT.PCR
*Fgf21*	ACCTCTACACGGATGATGCC	CCATCCTCAAGAAGCAGCTC		RT.PCR
*Pkm1*	ACCGCAAGCTGTTTGAAGAA	TCCATGAGGTCTGTGGAGTG		
*Pkm2*	GAGGCCTCCTTCAAGTGCT	CCAGACTTGGTGAGGACGAT		
*Myh7*	AAGGGCTTGAACGAGGAGTAGA	TTATTCTGCTTCCTCCAAAGGG	Wimmers *et al*.^59^	Annealing temperature 57 °C
*Myh2*	GCTGAGCGAGCTGAAATCC	ACTGAGACACCAGAGCTTCT		
*Myh1*	AGAAGATCAACTGAGTGAACT	AGAGCTGAGAAACTAACGTG		
*Myh4*	ATGAAGAGGAACCACATTA	TTATTGCCTCAGTAGCTTG		
*Eno3*	CATGAGGATTGAGGAGGCTCTT	GGCCTTTGGGTTACGGAACT		
*Idh2*	TTCATCAAGGAGAAGCTCATCCT	TGGTCTGGTCCCGGTTTG		
