# Supplementary figures and images for: Mitochondrial phosphoenolpyruvate carboxykinase (PEPCK-M) and serine biosynthetic pathway genes are co-ordinately increased during anabolic agent-induced skeletal muscle growth
Source: Sci Rep. 2016 Jun 28;6:28693. doi: 10.1038/srep28693 (PMC4923900; doi:10.1038/srep28693)

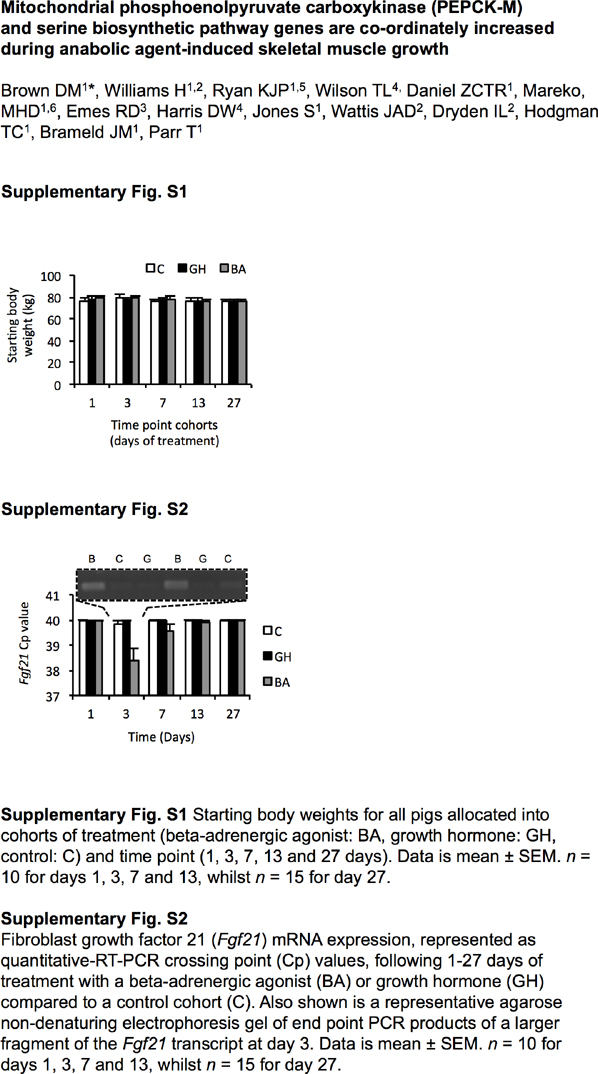

Supplement: Supplementary Information [file srep28693-s1.jpg]
